# Exposure to psychotropic drugs and breast cancer risk in patients with bipolar disorder and major depressive disorder: a nested case–control study

**DOI:** 10.1007/s00406-024-01798-9

**Published:** 2024-03-30

**Authors:** Dian-Jeng Li, Shih-Jen Tsai, Tzeng-Ji Chen, Chih-Sung Liang, Mu-Hong Chen

**Affiliations:** 1 Department of Addiction Science, Kaohsiung Municipal Kai-Syuan Psychiatric Hospital, Kaohsiung, Taiwan; 2 Department of Nursing, Meiho University, Pingtung, 91200 Taiwan; 3 Department of Psychiatry, Taipei Veterans General Hospital, No. 201, Sec. 2, Shihpai Road, Beitou District, Taipei, 11217 Taiwan; 4 Department of Psychiatry, College of Medicine, National Yang Ming Chiao Tung University, Taipei, Taiwan; 5 Department of Family Medicine, Taipei Veterans General Hospital, Taipei, Taiwan; 6 Institute of Hospital and Health Care Administration, National Yang Ming Chiao Tung University, Taipei, Taiwan; 7 Department of Psychiatry, Beitou Branch, Tri-Service General Hospital, Beitou District, No. 60, Xinmin Road, Taipei, 11243 Taiwan; 8 Department of Psychiatry, National Defense Medical Center, Taipei, Taiwan

**Keywords:** Breast neoplasm, Psychotropic drug, Bipolar disorder, Major depressive disorder, Case–control study

## Abstract

Breast cancer is one of the most prevalent and serious types of cancer globally. Previous literature has shown that women with mental illness may have an increased risk of breast cancer, however whether this risk is associated with the use of psychotropic drugs has yet to be elucidated. This study aimed to assess such risk among women with major depressive disorder (MDD) and bipolar disorder (BD). A nested case–control study design was used with data obtained from the Taiwan National Health Insurance Research Database. Logistic regression analysis with adjustments for demographic characteristics, medical and mental comorbidities, and all-cause clinical visits was performed to estimate the risk of breast cancer according to the cumulative defined daily dose (cDDD) of psychotropic drugs. The study included 1564 women with MDD or BD who had breast cancer, and 15,540 women with MDD or BD who did not have breast cancer. After adjusting for important confounders, the long-term use of valproic acid (odds ratio, 95% confidence interval: 0.58, 0.39–0.56, cDDD ≥ 365), citalopram (0.58, 0.37–0.91, cDDD 180–365), and sertraline (0.77, 0.61–0.91, cDDD ≥ 365) was associated with a lower risk of breast cancer compared to a cDDD < 30. The short-term use of fluvoxamine (0.82, 0.69–0.96, cDDD 30–180), olanzapine (0.54, 0.33–0.89, cDDD 30–179), risperidone (0.7, 0.51–0.98, cDDD 30–179), and chlorpromazine (0.48, 0.25–0.90, cDDD 30–179) was associated with a lower risk of breast cancer. We found no evidence of an increased risk of breast cancer in patients with MDD or BD receiving psychotropic drugs.

**Supplementary Information:**

The online version contains supplementary material available at 10.1007/s00406-024-01798-9.

## Introduction

Breast cancer is common among women worldwide [1], and previous research has suggested an increased risk of cancer mortality in patients with mental illness [2]. Despite this increased risk, the rate of cancer screening in such patients is often lower compared with the general population [3]. Several studies have also reported the under-utilization of preventive services, limited access to treatment, and challenges with adherence to cancer treatment in patients with mental illness [4, 5].

Findings regarding the association between cancer and major depressive disorder (MDD) and bipolar disorder (BD) are still controversial. One genetic study reported a significant risk of breast cancer in patients with MDD [6], while another study did not [7]. Due to the complicated relationship between breast cancer and mental illness, researchers have investigated the potential role of psychotropic drugs in this association, including antipsychotics, mood stabilizers, and antidepressants [8–11]. However, evidence from these studies is limited or inconclusive. Two large observational studies reported a significant risk of breast cancer associated with prolactin-related antipsychotics [10, 12], however whether prolactin-related antipsychotics stimulate breast cancer cell growth remains inconclusive [13, 14]. A critical review of human prospective studies showed equivocal results, with risk ratios ranging from 0.70 to 1.9 for premenopausal women and 0.76 to 2.03 for postmenopausal women [15]. Controversial findings have also been reported in the association between antidepressants and breast cancer risk. Several studies suggested that antidepressant use was linked to a 50–75% increased risk of breast cancer [16, 17], whereas another population-based study found no evidence of such an association [8]. As for other psychotropic drugs, numerous in vitro and in vivo preclinical studies have suggested that anticonvulsant drugs significantly inhibit cancer cell proliferation by modulating multiple signaling pathways. However, these effects have been demonstrated only in in vitro experiments using valproic acid [11], and evidence for carbamazepine [18] and lamotrigine [19] remains at the preclinical stage.

## Aim of the current study

There is currently insufficient evidence regarding the association between breast cancer and psychotropic drugs, especially mood stabilizers. In addition, the inconsistent results regarding antipsychotics and antidepressants highlights the need for further studies to better validate these findings. Besides research focusing on participants with schizophrenia [10], the association between the use of psychotropic agents and the risk of breast cancer has rarely been investigated among patients with BD or MDD. Given the gaps in previous evidence, the aim of this study was to comprehensively assess the risk of breast cancer associated with the prescription of psychotropic drugs (antipsychotics, antidepressants, and mood stabilizers) among a large cohort of patients with MDD and BD. Investigating this association could be helpful in clarifying the complex etiology of breast cancer risk among patients with severe mental illness (SMI), as well as providing healthcare workers with clinical implications for making informed decisions about the use of psychotropic drugs and discussing treatment options with patients. Our hypothesis is that certain psychotropic drugs may be associated with the risk of breast cancer.

## Methods

### Data source

The Taiwan National Health Research Institute oversees the National Health Insurance Research Database (NHIRD), which is available for scientific and research purposes [20, 21]. The NHIRD anonymizes individual medical records to protect patient privacy. In this study, we linked two databases: the specialized dataset of mental disorders, which includes all medical records (mental and non-mental) of insured individuals with mental disorders, and the Catastrophic Illness database, which includes diagnoses of catastrophic illnesses (such as malignant cancers) and the diagnosis date [22]. In Taiwan, the diagnosis of malignant cancers is reviewed by commissioned expert panels, and patients diagnosed with cancer are exempt from medical copayments. The diagnostic codes used in the NHIRD between 1996 and 2011 were based on the International Classification of Diseases, 9th Revision, Clinical Modification (ICD-9-CM). The Institutional Review Board of Taipei Veterans General Hospital approved the study protocol, and the requirement for informed consent was waived. The NHIRD has been used in many epidemiological studies in Taiwan [23–26].

### Study participants

**Fig. 1:**
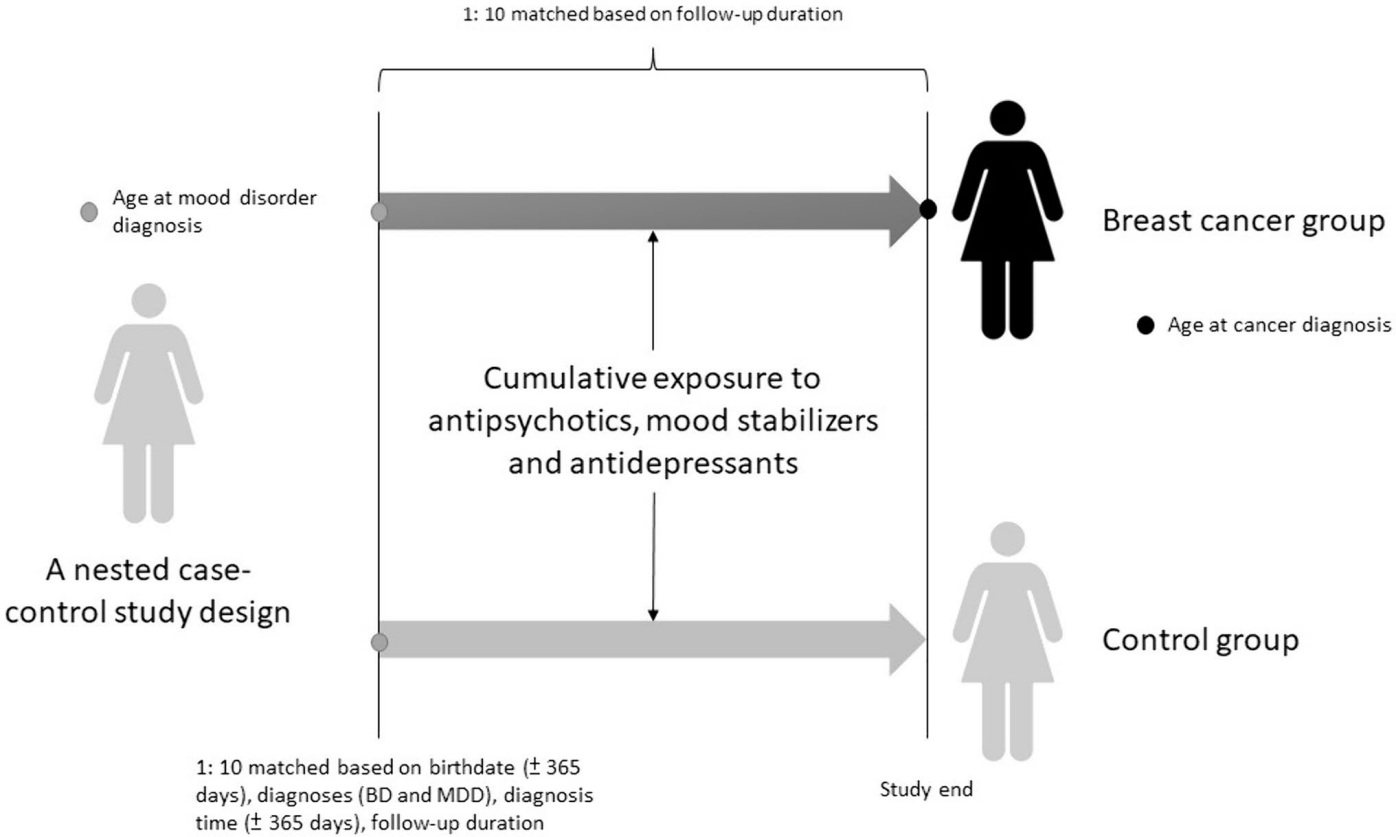
Study design illustration. *BD*: bipolar disorder, *MDD* major depressive disorder

The current study was designed as a nested case–control study. The study cohort consisted of women aged ≥ 20 years with psychiatrist-diagnosed BD (ICD-9-CM codes: 296.0x, 296.1x, 296.4x, 296.5x, 296.6x, 296.7x, 296.80, 296.81, 296.89) or MDD (ICD-9-CM codes: 296.2 × and 296.3x) who had no history of any malignant cancer prior to 2001 and up to 2011. The date of the diagnosis of BD or MDD was defined as the enrollment date, while the date of the diagnosis of breast cancer was defined as the endpoint date. The follow-up period was calculated from the enrollment date to the endpoint date. Cases were those who were subsequently diagnosed with malignant breast cancer (ICD-9-CM code: 174) from enrollment to the end of 2011 or death. Controls were selected from the cohort with BD or MDD but without a diagnosis of any malignant cancer. The controls were selected in a 10:1 ratio with the study cases based on birthdate (± 365 days), diagnosis (BD and MDD), diagnosis date (± 365 days), follow-up duration, medical and mental comorbidities (hypertension, dyslipidemia, diabetes mellitus, obesity, smoking, and alcohol and substance use disorders), income, and residence. A 10:1 ratio was used to enhance the statistical power and to ensure an adequate number of cases with BD or MDD for stratified analyses [27]. Income level (levels 1–3 per month: ≤ 19,100 New Taiwan Dollars [NTD], 19,001 ~ 42,000 NTD, and > 42,000 NTD) and urbanization level of residence (levels 1–5, most to least urbanized) were assessed as proxies for healthcare availability in Taiwan [28]. Additionally, data on Charlson Comorbidity Index (CCI) and all-cause clinical visits were obtained for the study and matched-control cohorts. The CCI, consisting of 22 physical conditions (cancer was excluded in the current study), was assessed to determine the systemic health conditions of the enrolled women [29]. In the CCI, each physical condition has an associated weight (from 1 to 6) based on the adjusted risk of mortality. The sum of all the weights yields a single comorbidity score for a patient. A score of zero indicates the absence of comorbidities, and the higher the score, the more likely the outcome will result in mortality or greater use of healthcare resources. The study design is shown in Fig. 1.

### Exposure to psychotropic medications

Information on prescribed drugs, including the World Health Organization Anatomical Therapeutic Chemical classification system, drug dosage, supply days, and number of dispensed drug pills, was extracted from the NHIRD. The World Health Organization’s defined daily dose (DDD) is a unit for measuring a prescribed amount of drug. The DDD is the assumed average maintenance dose per day of a drug consumed for its main indication. We calculated the cumulative DDD (cDDD) of all first-generation antipsychotics (FGAs; chlorpromazine, clotiapine, flupentixol, fluphenazine, haloperidol, loxapine, and sulpiride), second-generation antipsychotics (SGAs; aripiprazole, risperidone, olanzapine, amisulpride, ziprasidone, clozapine, and quetiapine), mood stabilizers (lithium, carbamazepine, oxcarbazepine, valproate, lamotrigine, topiramate, and gabapentin), and antidepressants (fluoxetine, sertraline, paroxetine, fluvoxamine, citalopram, escitalopram, venlafaxine, duloxetine, milnacipran, bupropion, and mirtazapine) during the follow-up period. Based on the cDDD [30–32], medication use patterns were classified into four subgroups: cDDD of < 30, cDDD of 30 to 179, cDDD of 180 to 364, and cDDD of ≥ 365.

### Statistical analysis

The independent t-test was used to compare continuous variables between groups, and Pearson’s χ^2^ test was used for nominal variables, as appropriate. Logistic regression analyses were performed with adjustments for demographic characteristics, medical and mental comorbidities, CCI scores, and all-cause clinical visits to calculate the odds ratio (OR) and 95% confidence interval (CI) of the association between psychotropic medication use (cDDD categories: < 30, 30–179, 180–364, and ≥ 365) and subsequent breast cancer risk. The psychotropic medications included FGAs, SGAs, mood stabilizers, and antidepressants. Previous studies have shown a slightly increased risk (incidence rate ratios ranging from 1.05 to 1.20) of breast cancer among women with major affective disorders, including BD and MDD [33–35]. Using G-power to estimate the sample size with an α error probability of 0.05 and a 1-β error probability (power) of 0.95, the optimal sample size was ≥ 10,905 at a rate ratio of 1.05, and ≥ 780 at a rate ratio of 1.20. A two-tailed p value < 0.05 was considered statistically significant. All data processing and statistical analyses were performed using Statistical Analysis Software (SAS) version 9.1 (SAS Institute, Cary, NC).

### Data availability statement

The NHIRD is released and audited by the Department of Health and Bureau of the NHI Program for scientific research purposes (https://nhird.nhri.org.tw/). The NHIRD can be obtained through a formal application regulated by the Department of Health and Bureau of the NHI Program.

## Results

### Demographic and clinical information

A total of 1564 cases (women with BD/MDD and breast cancer; BD = 283 and MDD = 1281) and 15,540 matched controls (women with BD/MDD without breast cancer) were included in the current study (Table 1). Compared to the control group, the breast cancer group had a significantly higher CCI score (2.68 vs 1.13, p < 0.001) and a higher number of all-cause clinical visits (19.62 vs 16.84, p < 0.001). However, the two groups did not significantly differ in terms of medical comorbidities, mental comorbidities, and demographic characteristics (Table 1). The distribution of antipsychotic prescriptions is listed in Supplementary Table 1.

**Table 1:** Demographic data between patients with and without breast cancer

	Patients with breast cancer (n = 1564)	Patients without breast cancer (n = 15,640)	p-value
Age at mood disorder diagnosis (years, SD)	50.42 (11.90)	50.42 (11.91)	0.999
Age at cancer diagnosis (years, SD)	54.59 (11.79)		
Follow-up duration (years, SD)	4.17 (2.17)	4.14 (2.61)	0.702
Diagnosis (n, %)			> 0.999
Bipolar disorder	283 (18.1)	2830 (18.1)	
Major depressive disorder	1281 (81.9)	12,810 (81.9)	
Medical and mental comorbidities (n, %)			
Hypertension	546 (34.9)	5540 (35.4)	0.697
Dyslipidemia	394 (25.2)	4109 (26.3)	0.365
Diabetes mellitus	322 (20.6)	3033 (19.4)	0.255
Obesity	69 (4.4)	681 (4.4)	0.903
Smoking	35 (2.2)	393 (2.5)	0.547
Alcohol use disorder	69 (4.4)	812 (5.2)	0.208
Substance use disorder	87 (5.6)	967 (6.2)	0.347
CCI score (SD)	2.68 (1.66)	1.13 (1.46)	< 0.001
Level of urbanization (n, %)			> 0.999
1 (most urbanized)	402 (25.7)	4020 (25.7)	
2	548 (35.0)	5480 (35.0)	
3	148 (9.5)	1480 (9.5)	
4	156 (10.0)	1560 (10.0)	
5 (most rural)	310 (19.8)	3100 (19.8)	
Income-related insured amount			> 0.999
≤ 15,840 NTD/month	837 (53.5)	8370 (53.5)	
15,841 ~ 25,000NTD/month	440 (28.1)	4400 (28.1)	
≥ 25,001NTD/month	287 (18.4)	2870 (18.4)	
All-cause clinical visits (times per year, SD)	19.62 (18.01)	16.84 (16.44)	< 0.001

*SD* standard deviation, *NTD* new Taiwan dollar, *CCI* Charlson Comorbidity Index

### Risk of breast cancer in different groups of exposure to mood stabilizers and antidepressants in the patients with BD or MDD

After adjusting for date of birth, demographic characteristics, date of diagnosis, follow-up duration, medical and mental comorbidities, CCI score, and all-cause clinical visits (Table 2), the group with a cDDD of ≥ 365 for all mood stabilizers was associated with a lower risk of breast cancer than the group with a cDDD of < 30 for all mood stabilizers (reported as OR with 95% CI 0.75; 0.59–0.95). Specifically, the group with a cDDD ≥ 365 of valproic acid was associated with a lower risk of breast cancer than the group with a cDDD of < 30 of valproic acid (0.58; 0.39–0.56). The group with a cDDD of ≥ 365 for all antidepressants was associated with a lower risk of breast cancer than the group with a cDDD of < 30 for all antidepressants (0.79; 0.68–0.92). The group with a cDDD of 180 to 364 of citalopram was associated with a lower risk of breast cancer than the group with a cDDD of < 30 of citalopram (0.58; 0.37–0.91). The group with a cDDD of 30 to 179 of fluvoxamine was associated with a lower risk of breast cancer than the group with a cDDD of < 30 of fluvoxamine (0.58; 0.37–0.91). The group with a cDDD of ≥ 365 of sertraline (0.77; 0.61–0.97) and the group with a cDDD of 30 to 179 of sertraline (0.68; 0.55–0.82) were associated with lower risks of breast cancer than the group with a cDDD of < 30 of sertraline.

**Table 2:** Exposures to mood stabilizers and antidepressants and breast cancer risk in patients with bipolar disorder and major depressive disorder

Event (%)	OR (95% CI; P)	Event (%)	OR (95% CI; P)	Event (%)	OR (95% CI; P)	Event (%)	OR (95% CI; P)	Event (%)	OR (95% CI; P)	Event (%)	OR (95% CI; P)	Event (%)	OR (95% CI; P)
All mood stabilizers		Carbamazepine		Valproic acid		Gabapentin		Lamotrigine		Lithium		Oxcarbazepine	
1315 (9.0)	1 (ref)	1513 (9.1)	1 (ref)	1438 (9.1)	1 (ref)	1549 (9.1)	1 (ref)	1542 (9.1)	1 (ref)	1470 (9.1)	1 (ref)	1554 (9.1)	1 (ref)
120 (10.4)	0.05 (0.85–1.30)	26 (9.6)	0.78 (0.50–1.21)	70 (9.2)	0.93 (0.71–1.22)	9 (10.6)	0.72 (0.33–1.55)	14 (8.4)	0.68 (0.48–1.23)	39 (9.3)	0.74 (0.66–1.34)	5 (11.6)	0.83 (0.31–2.27)
	0.681		0.262		0.606		0.401		0.205		0.719		0.720
37 (8.5)	0.84 (0.59–1.21)	14 (15.9)	1.48 (0.78–2.82)	23 (8.0)	0.76 (0.48–1.20)	4 (20.0)	1.58 (0.48–5.25)	4 (9.1)	0.99 (0.34–2.87)	10 (6.2)	0.69 (0.35–1.37)	3 (23.1)	1.64 (0.40–6.70)
	0.359		0.234		0.240		0.452		0.978		0.287		0.490
92 (8.8)	**0.75 (0.59–0.95)**	11 (7.3)	0.57 (0.29–1.11)	33 (8.1)	**0.58 (0.39–0.56)**	2 (7.7)	0.59 (0.13–2.68)	4 (12.5)	1.13 (0.35–3.69)	45 (9.1)	0.81 (0.58–1.12)	2 (33.3)	3.14 (0.53–18.55)
	**0.017**		0.096		**0.007**		0.494		0.835		0.206		0.207
Topiramate		All antidepressants		Bupropion		Citalopram		Duloxetine		Escitalopram		Fluoxetine	
1549 (9.1)	1 (ref)	402 (9.1)	1 (ref)	1496 (9.0)	1 (ref)	1415 (9.2)	1 (ref)	1516 (9.0)	1 (ref)	1472 (9.0)	1 (ref)	1106 (9.1)	1 (ref)
12 (10.7)	0.96 (0.50–1.84)	417 (8.9)	0.95 (0.81–1.10)	48 (10.7)	1.10 (0.80–1.52)	82 (8.4)	0.79 (0.62–1.02)	26 (11.1)	1.13 (0.72–1.77)	52 (10.0)	1.05 (0.77–1.44)	216 (8.5)	**0.82 (0.69–0.90)**
	0.889		0.477		0.566		0.067		0.587		0.764		**0.014**
2 (9.1)	1.12 (0.25–5.16)	206 (9.0)	0.93 (0.77–1.12)	12 (10.3)	0.97 (0.51–1.82)	22 (5.9)	**0.58 (0.37–0.91)**	13 (12.9)	1.38 (0.75–2.55)	19 (9.9)	0.93 (0.56–1.55)	85 (8.9)	0.83 (0.64–1.06)
	0.880		0.459		0.914		**0.018**		0.300		0.788		0.138
1 (10.0)	0.85 (0.10–7.33)	539 (9.3)	**0.79 (0.68–0.92)**	8 (8.0)	0.67 (0.30–1.47)	45 (8.7)	0.77 (0.55–1.08)	9 (10.8)	1.14 (0.55–2.37)	21 (9.8)	0.80 (0.48–1.32)	157 (9.8)	0.86 (0.71–1.04)
	0.879		**0.002**		0.317		0.125		0.726		0.386		0.109
Fluvoxamine		Milnacipran		Mirtazapine		Paroxetine		Sertraline		Venlafaxine			
1479 (9.1)	1 (ref)	1550 (9.1)	1 (ref)	1249 (9.1)	1 (ref)	1301 (9.0)	1 (ref)	1273 (9.3)	1 (ref)	1319 (9.1)	1 (ref)	cDDD < 30	
45 (8.8)	0.95 (0.68–1.31)	7 (6.2)	0.95 (0.68–1.31)	70 (9.2)	0.55 (0.24–1.56)	114 (8.4)	0.84 (0.68–1.04)	132 (7.4)	**0.68 (0.55-.0.82)**	101 (8.2)	0.80 (0.64–1.00)	cDDD, ≥ 30, < 180	
	0.737		0.156		0.181		0.107		** < 0.001**		0.054	cDDD, ≥ 180, < 365	
14 (8.6)	0.88 (0.49–1.58)	5 (13.9)	0.88 (0.49–1.58)	24 (8.2)	1.93 (0.72–5.17)	55 (10.9)	1.19 (0.88–1.62)	61 (9.5)	0.87 (0.65–1.16)	34 (7.1)	0.69 (0.47–1.00)	cDDD ≥ 365	
	0.672		0.151		0.386		0.260		0.344		0.051		
26 (10.5)	0.89 (0.57–1.40)	2 (5.1)	0.89 (0.57–1.40)	41 (9.9)	0.53 (0.12–2.37)	94 (11.2)	1.01 (0.89–1.29)	98 (9.0)	**0.77 (0.61–0.97)**	110 (11.1)	1.02 (0.82–1.27)		
	0.619		0.409		0.499		0.910		**0.029**		0.858		

Bold indicates *p* < 0.05

Adjusted for demographic data, medical comorbidities, CCI scores and all-cause clinical visits

*cDDD* cumulative defined daily dose, *OR* odds ratio, *SGA* second-generation antipsychotics, *FGA* first-generation antipsychotics, *n.a.* not available

### Risk of breast cancer in different groups of exposure to antipsychotics in the patents with BD or MDD

In terms of SGAs, the group with a cDDD of 30–179 for all SGAs had a lower risk of breast cancer compared to the group with a cDDD of < 30 for all SGAs (0.58; 0.44–0.75). The group with a cDDD of 30–179 of olanzapine had a lower risk of breast cancer compared to the group with a cDDD of < 30 of olanzapine (0.54; 0.33–0.89). The group with a cDDD of 30–179 of risperidone had a lower risk of breast cancer compared to the group with a cDDD of < 30 of risperidone (0.70; 0.51–0.98). The group with a cDDD of 30–179 of chlorpromazine had a lower risk of breast cancer compared to the group with a cDDD of < 30 of chlorpromazine (0.48; 0.25–0.90). However, the group with a cDDD of 180–364 of ziprasidone had a higher risk of breast cancer compared to the group with a cDDD of < 30 of ziprasidone (4.70; 1.47–15.07) (see Table 3).

**Table 3:** Exposures to antipsychotics and breast cancer risk in patients with bipolar disorder and major depressive disorder

Event (%)	OR (95% CI; p)	Event (%)	OR (95% CI; P)	Event (%)	OR (95% CI; P)	Event (%)	OR (95% CI; P)	Event (%)	OR (95% CI; P)	Event (%)	OR (95% CI; P)
All SGA		Amisulpiride		Aripiprazole		Clozapine		Olanzapine		Quetiapine	
1378 (9.2)	1 (ref)	1547 (9.1)	1 (ref)	1552 (9.1)	1 (ref)	1552 (9.1)	1 (ref)	1517 (9.1)	1 (ref)	1467 (9.0)	1 (ref)
75 (7.0)	**0.58 (0.44–0.75)**	10 (7.7)	0.59 (0.29–1.21)	8 (7.5)	0.77 (0.36–1.63)	3 (4.1)	0.35 (0.11–1.15)	19 (6.5)	**0.54 (0.33–0.89)**	53 (9.2)	0.74 (0.54–1.02)
	** < 0.001**		0.149		0.488		0.83		**0.016**		0.063
41 (9.7)	0.91 (0.64–1.29)	1 (2.7)	0.22 (0.03–1.64)	4 (14.3)	1.34 (0.42–4.26)	2 (7.1)	0.78 (0.18–3.39)	10 (9.9)	0.94 (0.47–1.86)	19 (9.5)	0.78 (0.46–1.31)
	0.598		0.139		0.617		0.736		0.850		0.343
70 (10.1)	0.80 (0.61–1.05)	6 (10.9)	0.97 (0.40–2.39)	0 (0.0)	n.a	7 (14.0)	1.70 (0.72–4.01)	18 (13.5)	1.11 (0.63–1.95)	25 (11.4)	0.95 (0.60–1.49)
	0.111		0.951		n.a		0.230		0.726		0.806
Risperidone		Ziprasidone		All FGA		Chlorpromazine		Clotiapine		Flupentixol	
1482 (9.1)	1 (ref)	1548 (9.0)	1 (ref)	1305 (9.0)	1 (ref)	1549 (9.2)	1 (ref)	1539 (9.1)	1 (ref)	1468 (9.0)	1 (ref)
48 (9.0)	**0.70 (0.51–0.98)**	8 (13.3)	1.06 (0.48–2.36)	167 (9.6)	0.86 (0.71–1.03)	12 (6.5)	**0.48 (0.25–0.90)**	12 (7.2)	0.60 (0.31–1.16)	81 (10.9)	0.93 (0.71–1.21)
	**0.037**		0.882		0.106		**0.023**		0.128		0.572
17 (10.6)	1.16 (0.68–1.99)	6 (46.2)	**4.70 (1.47–15.07)**	46 (10.5)	1.09 (0.79–1.51)	1 (2.0)	0.19 (0.03–1.40)	8 (12.3)	1.24 (0.57–2.69)	8 (6.5)	0.62 (0.29–1.32)
	0.590		**0.009**		0.609		0.103		0.589		0.219
17 (10.0)	0.83 (0.48–1.44)	2 (10.5)	1.21 (0.27–5.39)	46 (7.8)	0.72 (0.52–1.01)	2 (3.0)	0.28 (0.07–1.20)	5 (6.8)	0.72 (0.28–1.84)	7 (10.9)	0.95 (0.40–2.26)
	0.508		0.804		0.054		0.086		0.492		0.910
Fluphenazine		Haloperidol		Loxapine		Sulpiride					
1564 (9.1)	1 (ref)	1523 (9.1)	1 (ref)	1559 (9.1)	1 (ref)	1449 (9.1)	1 (ref)	cDDD < 30			
1 (0.0)	n.a	25 (9.1)	0.65 (0.41–1.03)	2 (10.0)	0.95 (0.20–4.43)	77 (9.1)	0.85 (0.66–1.11)	cDDD, ≥ 30, < 180			
	n.a		0.065		0.942		0.233	cDDD, ≥ 180, < 365			
0 (0.0)	n.a	6 (7.9)	0.76 (0.30–1.91)	0 (0.0)	n.a	21 (9.6)	0.93 (0.57–1.53)	cDDD ≥ 365			
	n.a		0.555		n.a		0.777				
0 (0.0)	n.a	10 (9.6)	1.03 (0.52–2.05)	3 (33.3)	3.93 (0.82–18.91)	17 (7.3)	0.73 (0.43–1.22)				
	n.a		0.926		0.087		0.228				

Bold indicates *p* < 0.05

Adjusted for demographic data, medical comorbidities, CCI scores and all-cause clinical visits

*cDDD* cumulative defined daily dose, *OR* odds ratio, *SGA* second-generation antipsychotics, *FGA* first-generation antipsychotics, *n.a*. not available

## Discussion

### Main findings of the current study

The results of this study demonstrated that overall, the use of mood stabilizers, antidepressants, and SGAs was associated with a reduced risk of breast cancer among female patients with BD or MDD. Specifically, of the mood stabilizers, the long-term use of valproic acid was linked to a decreased risk of breast cancer. In addition, the long-term use of citalopram or sertraline was associated with a lower risk of breast cancer, while the short-term use of fluvoxamine, chlorpromazine, olanzapine, or risperidone was associated with a lower risk of breast cancer. However, the use of ziprasidone with a cDDD of 180 to 364 (not long-term use) was linked to an increased risk of breast cancer compared to a cDDD of < 30. In summary, we found that the use of the aforementioned psychotropic agents except ziprasidone may reduce the risk of breast cancer in patients with BD and MDD.

### Decreased risk of breast cancer with the use of mood stabilizers and antidepressants

Our results demonstrated that the long-term use of mood stabilizers was associated with a reduced risk of breast cancer in the study patients, and in particular the long-term use of valproic acid over the short-term use. A potential mechanism for this effect is that valproic acid, a broad class I histone deacetylase (HDAC) inhibitor, may decrease the expression of the pyruvate kinase M2 isoform, leading to inhibited cell proliferation and reduced colony formation in breast cancer cells [36]. Several in vitro and in vivo preclinical studies have suggested that valproic acid significantly inhibits cancer cell proliferation and metastasis by modulating multiple signaling pathways, tumor immune response, and cell cycle arrest through HDAC inhibition [11, 37].

The debate surrounding antidepressant treatment and increased risk of breast cancer has persisted for many years. While preliminary data have suggested a risk of breast cancer in users of antidepressants [16, 17], other epidemiological studies using nationwide databases [8] and large prospective surveys (Women’s Health Initiative Observational Study) [38] have found no such association. A possible etiology linking antidepressants to the risk of breast cancer may be through the effect of prolactin. Treatment with selective serotonin reuptake inhibitors (SSRIs) may increase circulating prolactin levels [39, 40], which could potentially increase the risk of breast cancer by stimulating cellular proliferation, differentiation, and angiogenesis [13]. However, recent evidence does not support the association between SSRIs and the risk of breast cancer [41], and a recent cohort study did not observe increased prolactin levels among women using antidepressants or SSRIs [42]. The findings of such an association in previous studies may be due to relatively small sample sizes or confounding factors. In contrast to previous findings, we found that the long-term use of antidepressants including citalopram, fluvoxamine, and sertraline may be associated with a decreased risk of breast cancer. The mechanism for this effect may be explained by the anti-inflammatory effects of antidepressants [43, 44], which may mitigate the hypothesized influences of anti-inflammatory agents on the risk of breast cancer [45]. In addition, the long-term use of antidepressants may also be beneficial in stabilizing mood patterns, and we hypothesize that patients who receive regular treatment for their mental illness may have a healthier lifestyle, which could reduce environmental risk factors for cancer. Furthermore, citalopram, sertraline, and fluvoxamine are relatively weaker inhibitors of CYP2D6 compared to stronger inhibitors such as paroxetine or fluoxetine [46]. Previous studies have suggested that CYP2D6 inhibitors such as paroxetine may impair the conversion of tamoxifen into its active form and hinder its efficacy in treating breast cancer [47, 48]. Taken together, these findings suggest the advantages of citalopram, sertraline, and fluvoxamine in either reducing the risk of breast cancer or the effect on tamoxifen. In addition to biological etiologies, the long-term use of mood stabilizers or antidepressants may also have therapeutic effects. Taking psychotropic agents regularly may improve mental health, and consequently patients may experience less stress and pay more attention to their physical health. Therefore, a balanced mental state (achieved through drug treatment) may have a positive effect on preventing the development of cancer.

### Association between the use of antipsychotics and risk of breast cancer

The long-term use (cDDD ≥ 365) of SGAs (OR: 0.8) and FGAs (OR: 0.72) showed a trend towards a decreased risk of breast cancer, while the short-term use (30–179) of SGAs overall, olanzapine, risperidone, and chlorpromazine was significantly associated with a decreased risk of breast cancer. These results differ from previous literature [10, 12] that reported an association between an increased risk of breast cancer and prolactin-related antipsychotics such as risperidone. Several factors may contribute to this discrepancy. First, the association between prolactin secretion and the incidence of breast cancer remains controversial [15]. While some studies have suggested that prolactin stimulates cellular proliferation, differentiation, and angiogenesis of breast cancer [13], other studies have not supported this hypothesis. There is also evidence suggesting that prolactin may have a protective effect and suppress breast cancer cell growth. A preclinical study demonstrated that prolactin could suppress cellular growth or metastasis of breast cancer [14], and another study indicated that the 16-kDa prolactin isoform, a prolactin fragment, had anti-angiogenic effects in in vivo experiments [49]. These findings suggest the potentially biological mechanism by which prolactin-related antipsychotics may reduce the risk of breast cancer. Second, treatment with antipsychotics may improve the symptoms of BD or MDD, which could explain our findings. An observational study of individuals with SMI reported that poorer insight and awareness of the illness were significantly associated with greater disease severity [50]. Previous reviews have shown that SMI is associated with the under-utilization of preventive services and less regular mammography [4, 5, 51]. Therefore, we hypothesize that antipsychotics can help to reduce the severity of illness, leading to improved awareness of self-health and early identification of breast masses during the precancer stage. Third, differences in the study populations may also play a role. Previous studies have recruited patients with schizophrenia [10], while we recruited patients with BD or MDD. Exposure to antipsychotics differs between schizophrenia and mood disorders. For instance, the recommended dose of risperidone for the adjunctive treatment of MDD is 1 to 3 mg per day [52], while the recommended dose for BD is 2 to 4 mg per day [53]. However, the recommended dose of risperidone for schizophrenia is 3 to 6 mg per day [54], indicating a higher cumulative daily dose of antipsychotics compared to BD or MDD. Further studies are needed to clarify the complicated etiologies behind the effect of different mental illnesses. Surprisingly, we found that ziprasidone increased the risk of breast cancer. To the best of our knowledge, no other study has investigated the association between ziprasidone and increased risk of breast cancer, although one preclinical study demonstrated that ziprasidone could suppress pancreatic adenocarcinoma cell proliferation, indicating a potentially anti-cancer effect [55]. However, the relatively wide confidence interval (1.47–15.07) suggests that this result may be confounded by statistical power or chance.

### Limitations

There are several limitations to the current study. First, some ORs could not be estimated due to differences in the logistic regression model, including fluoxetine. Second, due to limitations imposed by the institutional review board, the frequency of mammography could not be identified, however this may have affected the risk of breast cancer. Third, the CCI can only evaluate the potential effect of medical comorbidities, however it is difficult to ascertain to what extent these comorbidities affect breast cancer. Fourth, the NHIRD only includes residents in Taiwan, which may limit the generalizability of our findings to a more global context. Fifth, we combined patients with BD and patients with MDD in the analysis regarding the required sample size. Finally, although we adjusted for multiple factors related to the risk of breast cancer, residual confounding factors may still exist, which may have affected the associations between psychotropic drugs and the risk of breast cancer in our study.

## Conclusions

Our results showed that treatment with several mood stabilizers (valproic acid), antidepressants (citalopram, sertraline, and fluvoxamine), or antipsychotics (chlorpromazine, olanzapine, or risperidone) was associated with a decreased risk of breast cancer in female patients with BD or MDD. These findings provide valuable information for clinicians when discussing psychotropic drug options with patients who have MDD or BD, particularly those with other risk factors for breast cancer. However, the use of antipsychotics for patients with BD or MDD still needs to be decided based on the patient’s psychiatric symptoms and physical condition. Further neurobiological investigations are needed to better understand the underlying etiologies behind the association between the use of psychotropic drugs and breast cancer.

## Supplementary Information

Below is the link to the electronic supplementary material.

Supplementary file1 (DOCX 16 KB)

## Data Availability

Anonymized data, as described in this manuscript, will be shared upon request from any qualified investigator by the corresponding author (Dr. Mu-Hong Chen, email: kremer7119@gmail.com).
