# Supplementary material for: Exposure to psychotropic drugs and breast cancer risk in patients with bipolar disorder and major depressive disorder: a nested case–control study
Source: Eur Arch Psychiatry Clin Neurosci. 2024 Mar 30;275(2):533–43. doi: 10.1007/s00406-024-01798-9 (PMC11910431; doi:10.1007/s00406-024-01798-9)
Supplement: Supplementary file 1 — Supplementary file1 (DOCX 16 KB) [file 406_2024_1798_MOESM1_ESM.docx]

Supplementary table 1. Distribution of antipsychotics prescriptions among patients with major depressive disorder and bipolar disorder

|  | | | Group | | Total |
| --- | --- | --- | --- | --- | --- |
|  |  |  | MDD | BD |  |
| SGA | cDDD < 30 | Number | 13013 | 1999 | 15012 |
|  |  | % | 92.3% | 64.2% | 87.3% |
|  | cDDD, ≥ 30, < 180 | Number | 603 | 471 | 1074 |
|  |  | % | 4.3% | 15.1% | 6.2% |
|  | cDDD, ≥ 180, < 365 | Number | 191 | 231 | 422 |
|  |  | % | 1.4% | 7.4% | 2.5% |
|  | cDDD ≥ 365 | Number | 284 | 412 | 696 |
|  |  | % | 2.0% | 13.2% | 4.0% |
| Total | | Number | 14091 | 3113 | 17204 |
|  |  | % | 100.0% | 100.0% | 100.0% |

|  | | | Group | | Total |
| --- | --- | --- | --- | --- | --- |
|  |  |  | MDD | BD |  |
| FGA | cDDD < 30 | Number | 12493 | 1953 | 14446 |
|  |  | % | 88.7% | 62.7% | 84.0% |
|  | cDDD, ≥ 30, < 180 | Number | 1144 | 588 | 1732 |
|  |  | % | 8.1% | 18.9% | 10.1% |
|  | cDDD, ≥ 180, < 365 | Number | 237 | 202 | 439 |
|  |  | % | 1.7% | 6.5% | 2.6% |
|  | cDDD ≥ 365 | Number | 217 | 370 | 587 |
|  |  | % | 1.5% | 11.9% | 3.4% |
| Total | | Number | 14091 | 3113 | 17204 |
|  |  | % | 100.0% | 100.0% | 100.0% |

SGA: second-generation antipsychotics; FGA: first-generation antipsychotics; MDD: major depressive disorder; BD: bipolar disorder.
